# The Gini coefficient as a surrogate for the regulability or homeostasis of metabolite concentrations: focus on ergothioneine

**DOI:** 10.1007/s11306-026-02534-1

**Published:** 2026-09-16

**Authors:** Douglas B. Kell, Warwick B. Dunn, Catherine L. Winder, Keerthana Anand, Benjamin Greenfield, Louise C. Kenny, Abi Merriel, J. Bernadette Moore, Catriona Waitt

**Affiliations:** 1 Centre for Metabolomics Research, Department of Biochemistry, Cell and Systems Biology, Institute of Systems, Molecular and Integrative Biology, University of Liverpool, Crown St, Liverpool, L69 7ZB UK; 2 Department of Physiological Sciences, Faculty of Science, Stellenbosch University, Stellenbosch Private Bag X1, Matieland, 7602 South Africa; 3 Department of Women’s and Children’s Health, Faculty of Health and Life Sciences, University of Liverpool, Liverpool, L7 8TX UK; 4 Infectious Diseases Institute, Makerere University College of Health Sciences, Kampala, Uganda

## Abstract

The Gini coefficient is a simple non-parametric statistic that reflects the degree of variation of a property between individuals or samples, taking a value between zero and one. We here develop the idea that if the metabolism of a metabolite is tightly regulated it should have a low Gini coefficient whereas if for instance it is exogenous and present only in a subset of samples (e.g. from individuals that have ingested the substance and/or metabolised it to various degrees) the Gini coefficient will be much greater. Analysis of published datasets shows this to be true, allowing one to suggest that the Gini coefficient might assist in assessing both regulability and the origin of a particular metabolite. Plasma amino acid levels are especially tightly regulated (some have a Gini coefficient less than 0.1, a value lower than that for any transcript), while exogenous drugs and their metabolites have the highest Gini coefficients (some over 0.8). Vitamins are essential and exogenous (but regulated) and have intermediate values (ca. 0.2–0.4). Ergothioneine is a valuable nutraceutical that is not synthesised by humans and it is also of some interest to assess the variation in its distribution. A number of studies find it normally to have a Gini coefficient in the range 0.3–0.4, and with lower levels of the Gini coefficient (as in a pilot study summarised here) suggesting a relatively weak exogenous exposure. Overall, we consider that the Gini coefficient provides a convenient means of describing the extent to which a metabolite concentration varies over a metabolomic dataset, and hence the extent to which it may be regulated.

**Supplementary Information:**

The online version contains supplementary material available at https://doi.org/10.1007/s11306-026-02534-1.

## Introduction

Originally applied within economics (Ceriani & Verme, 2012; Gini, 1912; Wilkinson & Pickett, 2009, 2024), the Gini coefficient represents a convenient and non-parametric measure for describing the variation or ‘inequality’ in the distribution of a property (such as wealth or income). We have previously applied it to transcriptomics data (O’Hagan et al., 2018; Wright Muelas et al., 2019), including for the identification of suitable ‘housekeeping’ genes (Joshi et al., 2022) that might best be used for transcriptome normalisation (Wright Muelas et al., 2019). The Gini coefficient can take a value between zero (in the case, for transcriptomics, that each example has the same normalised RNA expression) and 1 (the gene is transcribed in only a single example). It has occasionally also been applied to describe the distributions of properties of populations of small molecules (Graczyk, 2007; Kumar & Bhatt, 2025; Ursu et al., 2020; Weidlich & Filippov, 2016).The idea that we seek to develop here is that if a metabolite is tightly regulated by a cell, tissue or organism it is likely to have a low Gini coefficient whereas if it is exogenous (e.g. a synthetic pharmaceutical drug or a foodstuff containing secondary metabolites) and present only in a subset of samples (here from individuals that have ingested the drug or food) the Gini coefficient will be much greater. The first purpose of this paper is to analyse suitable published datasets to assess the truth of this idea. We note that according to the principles of Metabolic Control Analysis (Fell, 1992; Heinrich & Rapoport, 1974; Kacser & Burns, 1973; Kell & Westerhoff, 1986) variations in the level of (a transcript or) an enzyme have small effects on metabolic fluxes but potentially large effects of metabolite concentrations. A priori it might then be expect that the Gini coefficients of metabolites might tend, if unregulated, to be rather greater than those of transcripts or proteins (Kell & Oliver, 2016; Oliver et al., 1998; Raamsdonk et al., 2001). Gini-type quantities have occasionally been used in metabolomics, but most commonly as random-forest feature importance (Grissa et al., 2016; Voges et al., 2023) or AUC-derived discrimination metrics (Bissan et al., 2025). However, we are not aware of any systematic use of the classical Lorenz/Gini coefficient to rank individual metabolites by the inequality of their plasma (or urine) concentrations across subjects as a surrogate for homeostatic regulation versus dietary, pharmaceutical or other exogenous exposure.

Ergothioneine is a nutraceutical that has attracted considerable interest (e.g. (Aruoma et al. 2012; Beelman et al. 2022; Borodina et al. 2020; Cheah and Halliwell 2012; Chen et al. 2024b; Fovet et al. 2022; Fu and Shen 2022; Fu et al. 2025; Halliwell and Cheah 2024; Halliwell et al. 2016, 2018, 2023; Han et al. 2021; Harasym et al. 2025; Kell et al. 2025; Kitsanayanyong and Ohshima 2021; Lei et al. 2025; Liu et al. 2023; May-Zhang et al., 2025; Nakamichi et al., 2022; Nguyen et al., 2013; Roberts et al., 2026; Servillo et al., 2017; Smith et al., 2020; Takhor & Phan, 2025; Thomas et al., 2024; Tian et al., 2023; Wen et al., 2025; Wijesekara & Xu, 2024; Yau et al., 2024; Zhang et al., 2025) as higher concentrations have been shown to be associated with a lower incidence of a variety of cardiovascular diseases (e.g. (Kameda et al. 2020; Kell et al. 2025; Smith et al. 2020; Wu et al. 2021b). In an example from our own studies based on its role in preventing pre-eclampsia (see (Ho, 2023; Kell et al., 2025; Kenny et al., 2023; Roberts et al., 2026), its distribution across individuals in a population was shown to be far from normal in the statistical sense, something that is widely observed in metabolomics (Antonelli et al., 2019; Ghosh et al., 2024; Lu & King, 2009).

In view of these potential protective effects of ergothioneine, it was of further interest to assess distributions of its concentration that have been observed experimentally using mass spectrometry. Ergothioneine (PubChem CID 5351619), with a molecular formula of C_9_H_15_N_3_O_2_S, has an exact monoisotopic mass of 229.0885 Da; in ESI+ mode the protonated form [M + H]^+^ has a monoisotopic mass of 230.0958 m/z (229.0885 + 1.0073) while the *m/z* in ESI- mode is 228.0812 ([M-H]^−^). Fortuitously, no other biologically relevant molecules lies within 3ppm of this (or even 10 ppm), which makes searching databases for ergothioneine rather straightforward, even if it is not identified explicitly. Thus a second purpose of this paper was to assess the values of its Gini coefficient observed in experimental studies. Since ergothioneine is not synthesised by mammals and is therefore exogenous in origin, it was to be assumed that it would be an example of a molecule with a relatively ‘high’ Gini coefficient, allowing one to establish what ‘high’ might mean in this context. In the case of transcriptome data, ‘low’ Gini coefficients were < 0.2, though they were few and far between (O’Hagan et al., 2018; Wright Muelas et al., 2019).

## Methods

### Publicly available datasets

These were downloaded from the sources recorded below. Data analyses were performed using Python 3.12.7. Probability Density Function (PDF) and Cumulative Distribution Functions (CDF), as well as other statistics, were calculated using the scipy.stats package (1.16.2). 95% confidence intervals were caluclated from 2000-fold boorstrapping. Gini coefficients were calculated using the algorithm built in to NumPy. Note that the Gini coefficient is completely insensitive to normalisation, and that if some values are zero they are treated as such. Some data were plotted using Spotfire (https://spotfire.com/).

### Ergothioneine study

#### Sample collection

New data presented include a targeted UHPLC-MS metabolite dataset collected from patients booked for their pregnancies at Liverpool Women’s hospital in 2025 of 40 antenatal samples from 10 women with a diagnosis of pre-eclampsia and 30 matched controls (one of whom went onto develop pre-eclampsia). Ethical approvals for the collection of these data was obtained from the South West- Frenchay Research Ethics Committee on 4th February 2025 (24/SW/0155). Samples were collected into a sodium citrate tube after obtaining informed consent. Once taken they were gently inverted six times before transport to the laboratory where they were left for 30 min prior to centrifuging at 3000 x g for 15 min. The supernatant was collected and stored at −80 °C prior to processing.

##### Chemicals

Ergothioneine (purity 99.67%) and water (LC-MS Grade; LiChrosolv) were purchased from Merck (Gillingham, UK). Methanol, (> 99.9% HiPerSolve CHROMANORM) and acetonitrile (> 99.9% HiPerSolve CHROMANORM) were purchased from VWR (Bristol, UK).

#### Sample extraction

Samples were randomised using the RAND() function in Microsoft Excel to determine the sample preparation order. Serum samples were thawed on ice on the day of preparation. 50 µL of serum and 150 µL acetonitrile/methanol (1:1, v/v) were mixed by vortex (120 s) and centrifuged (17,000 *x g*, 20 min, 4 °C). The supernatant was transferred to an LC vial ready for analysis. Blank extraction solutions were prepared by performing the extraction protocol as described above for samples with no serum present.

#### Preparation of calibration solutions of ergothioneine

A stock solution of concentration 1000 µmol.L^− 1^ was prepared in water/acetonitrile/methanol (25.0/37.5/37.5, v/v/v). Serial dilution of this stock solution was performed using the same solvent to prepare calibration solutions at the following concentrations – 0.01, 0.1, 0.5, 1.0, 5.0, 10.0, 25.0, 50.0, 100.0, 200.0, 300.0, 400.0 and 500.0 µmol.L^− 1^.

#### Ultra-high performance liquid chromatography-mass spectrometry (UHPLC-MS)

The samples (maintained at 4 °C) were analysed using a Vanquish binary pump H system coupled with a heated electrospray Orbitrap Exploris 240 mass spectrometer (Thermo Fisher Scientific, MA, USA). Each biological sample was injected once. Sample extracts were analysed using an Accucore-150-Amide-HILIC column (100 × 2.1 mm, 2.6 μm, Thermo Fisher Scientific, MA, USA). For positive ion mode mobile phase A was 95% acetonitrile/water (10mM ammonium formate, 0.1% formic acid) and mobile phase B was 50% acetonitrile/water (10 mM ammonium formate, 0.1% formic acid). The gradient elution applied was: t = 0.0, 1% B; t = 2.1, 1% B; t = 4.6, 15% B; t = 7.6, 50% B; t = 10.1, 95% B; t = 11.0, 95%B; t = 11.5, 1%B, t = 15.0, 1% B. All changes were linear (curve = 5) and the flow rate was 0.50 mL.min^− 1^. Column temperature was 35 °C and injection volume was 2µL. Data were acquired in positive ionisation mode in the m/z range of 76 − 1050 with a mass resolution of 120,000 (FWHM at m/z 200). Ion source parameters applied were: sheath gas = 40 arbitrary units, aux gas = 8 arbitrary units, sweep gas = 1 arbitrary units, spray voltage = 3.2 kV (positive ion mode) and 2.7 kV (negative ion mode), vaporizer temperature = 320 °C and ion transfer tube temperature = 250 °C. All samples were collected as MS1 data in the profile mode applying scan time = 256ms, microscans = 1, RF lens = 50% and normalised AGC target = 100%. Orbitrap iExploris 240 Tune application software controlled the instrument. Two blank samples and one replicate of all concentration calibration solutions (from 0.01 to 1000 µmol.L^− 1^) were analysed first followed by analysis of all 40 serum samples in a randomised order.

#### Data processing

The data were processed using ThermoFisher Scientific TraceFinder version 5.1 (Thermo Fisher Scientific, MA, USA), employing the TargetPeak workflow to detect Ergothioneine [M + H]^+^ adduct with a mass tolerance of 5ppm and retention time tolerance of 30 s. A calibration curve was constructed using peak area data for ergothioneine plotted against concentration. The calibration curve was applied to determine the ergothioneine concentration in serum samples.

## Results

### Use of the Gini coefficient for describing the spread of values of a variable in a population of samples

As noted above, we have found the Gini coefficient, that takes a value between 0 and 1, to be a convenient measure of the variation of a transcriptome distribution (O’Hagan et al., 2018; Roberts et al., 2026; Wright Muelas et al., 2019). We surmise that its value will be low for a metabolite that is tightly controlled, but larger for exogenous drugs or foodstuffs (and their endogenously modified metabolic products) that are not necessarily ingested by everyone in a population and whose concentrations consequently depend mainly on pharmaceutical or dietary exposure.

**Fig. 1:**
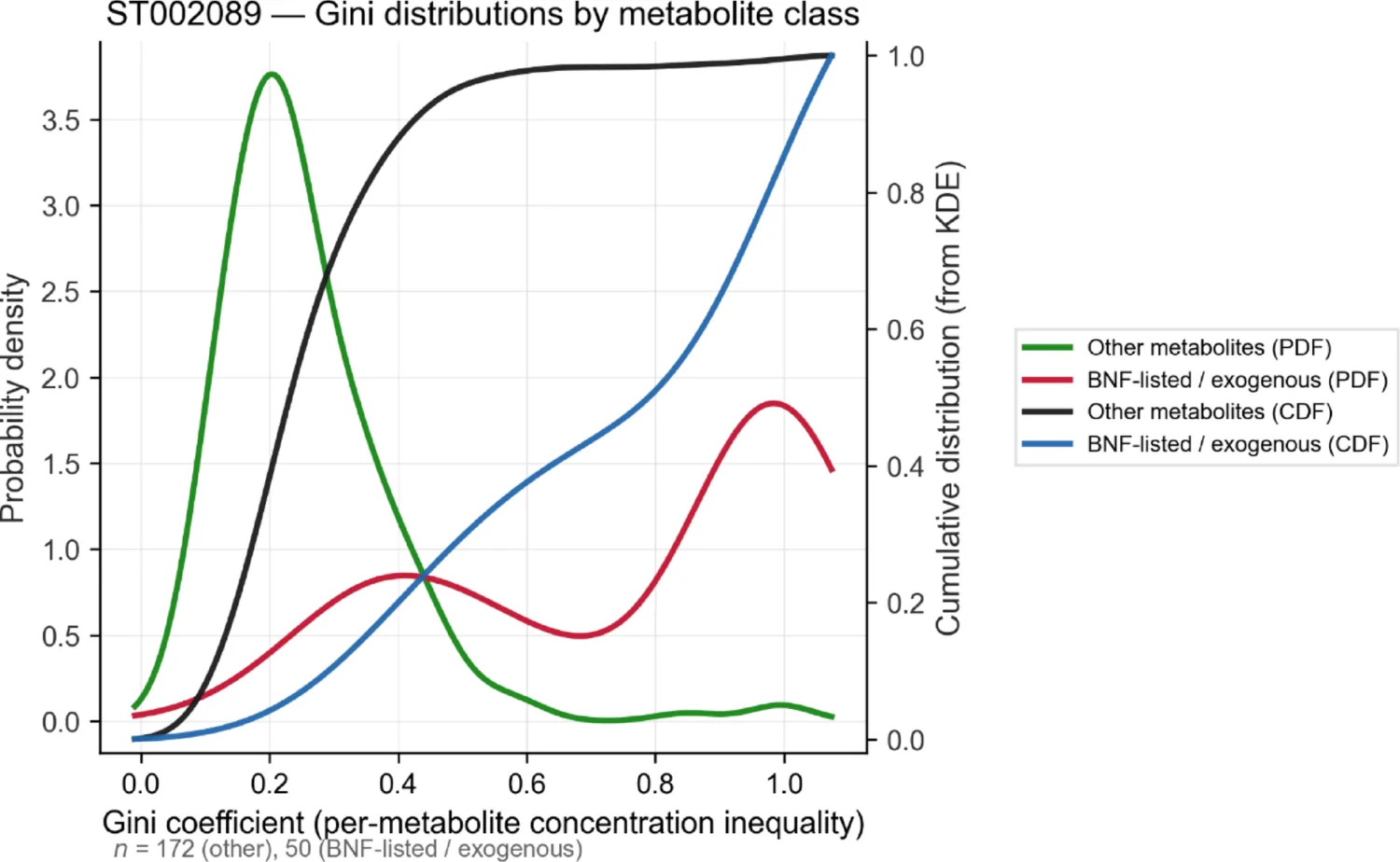
Smoothed probability density and cumulative distribution functions for the ES- metabolites listed in Metabolomics Workbench ST002089 from the study of Gillenwater and colleagues (Gillenwater et al., 2021). “BNF-listed/exogenous” metabolites are those tagged as such in our Supplementary Table 1. Nominally endogenous metabolites are the others

**Fig. 2:**
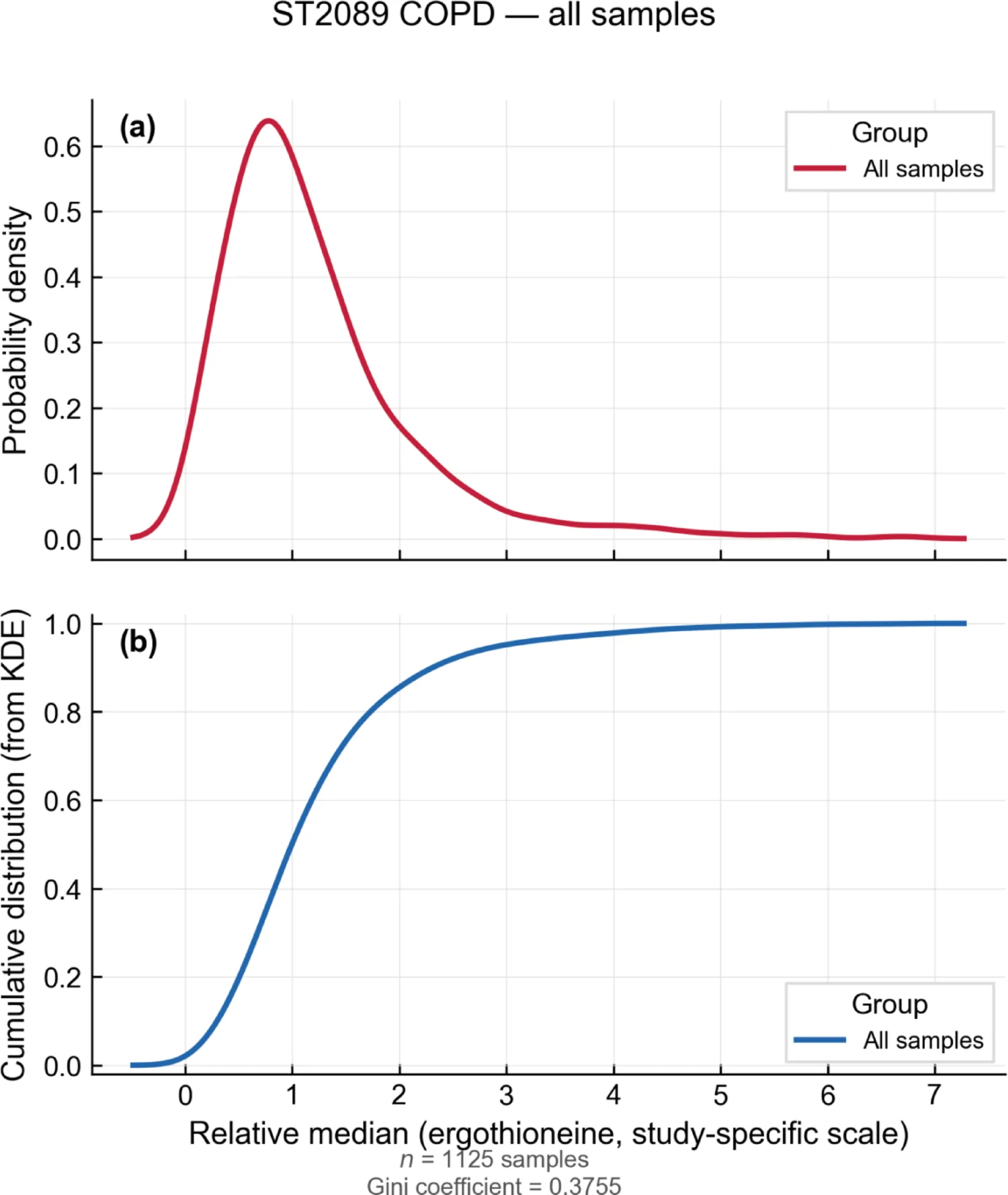
Smoothed probability density and cumulative distribution functions for ergothioneine in the COPD dataset (Metabolomics Workbench ST002089) from the study of Gillenwater and colleagues (Gillenwater et al., 2021)

Here we do not need absolute concentrations, just relative signals, for within-study comparisons. Thus, in some cases there are excellent data illustrating distributions, but they were not convertible into absolute concentrations. One such case is an extensive study of over 200 identified metabolites in plasma in ESI+ mode for 1125 individuals with chronic obstructive pulmonary disease (Gillenwater et al., 2021) (Metabolomics Workbench dataset ST002089) (see (Sud et al., 2016), for each of which we calculated their Gini coefficient. Figure 1 shows the probability density and cumulative distribution functions for these Gini coefficient data. Molecules were labelled by the authors as drugs if they were known to be of pharmaceuticals/foodstuff origins but as metabolites if they were likely to be ingested by a majority (including the essential amino acids, given that every sensible diet must include protein). In the event, the Gini coefficients for both essential and non-essential amino acids were identical and averaged just 0.14. In fact nine of the 25 lowest Gini coefficients were amino acids (M, R,P, S,F, N,W, K,Q). Where we could not define the class these were removed (5 cases). The distinction is very clear, lending credence to the idea that the Gini coefficient reflects the regulability or extent of homeostasis of the metabolite of interest. Thus the peak Gini coefficient in the probability density distributions was ~ 0.2 for endogenous metabolite (‘endogenites’ (O’Hagan & Kell, 2015, 2016) but more than 0.95 for exogenous molecules. The median Gini coefficient was 0.263. In this dataset ergothioneine had a Gini coefficient of 0.38 (Fig. 2). The percentage of molecules in the two classes with Gini coefficient above 0.5 is 73% for exogenous molecules and just 2.9% for molecules considered endogenous or to be regulated. The full analysis is provided in Supplementary Table 1.

### Vitamins

Vitamins are of course, by definition, of exogenous origin. We downloaded data from various studies on vitamin concentrations at NHANES (e.g. for vitamin D the URL is https://wwwn.cdc.gov/Nchs/Data/Nhanes/Public/2021/DataFiles/VID_L.htm) and converted the data held there in xpt format to csv format using the SAS Universal Viewer, and then the Gini coefficient was calculated. Table 1 summarises the results.

**Table 1:** Gini coefficient for the variation in vitamin concentrations in erythrocytes (folate) or serum (other vitamins)

File	NHANES cycle	Individuals (rows)	Primary analyte (Gini variable)	Valid *n*	Gini
FOLATE_L	Aug 2021– Aug 2023	8,727	RBC folate LBDRFOSI (nM)	7,506	0.225
VID_L	Aug 2021– Aug 2023	8,727	Total 25(OH)D LBXVIDMS (nM)	7,307	0.251
VIC_J	2017– Mar 2020	7,435	Vitamin C LBDVICSI (µM)	6,740	0.291
VITAEC_J	2017– Mar 2020	7,435	α-Tocopherol LBDVIESI (µM)	6,665	0.189
VITB12_H	2013–2014	5,736	Vitamin B12 LBDB12SI (pM)	5,447	0.326
VIT_B6_F	2009–2010	9,835	PLP LBXPLP (nM)	8,431	0.425

Interestingly, although these are exogenous metabolites, they are significantly more regulated than are some of the other exogenous metabolites. Indeed, as cofactors, vitamins are often recognised as part of ‘currency metabolites’ (see e.g. (Gerlee et al., 2009; Kell & Mendes, 2000) and thereby tend to be more tightly regulated than are other exogenous substances (e.g. (Chen et al. 2024a; Sun et al. 2023). This said, the Gini coefficient for these vitamins in serum varies more than threefold, leaving open the question as to why. We note that vitamin status reflects a convolution of diet, absorption, host metabolism, supplementation and microbiome interactions, and again these are rarely recorded in the relevant studies.

**Fig. 3:**
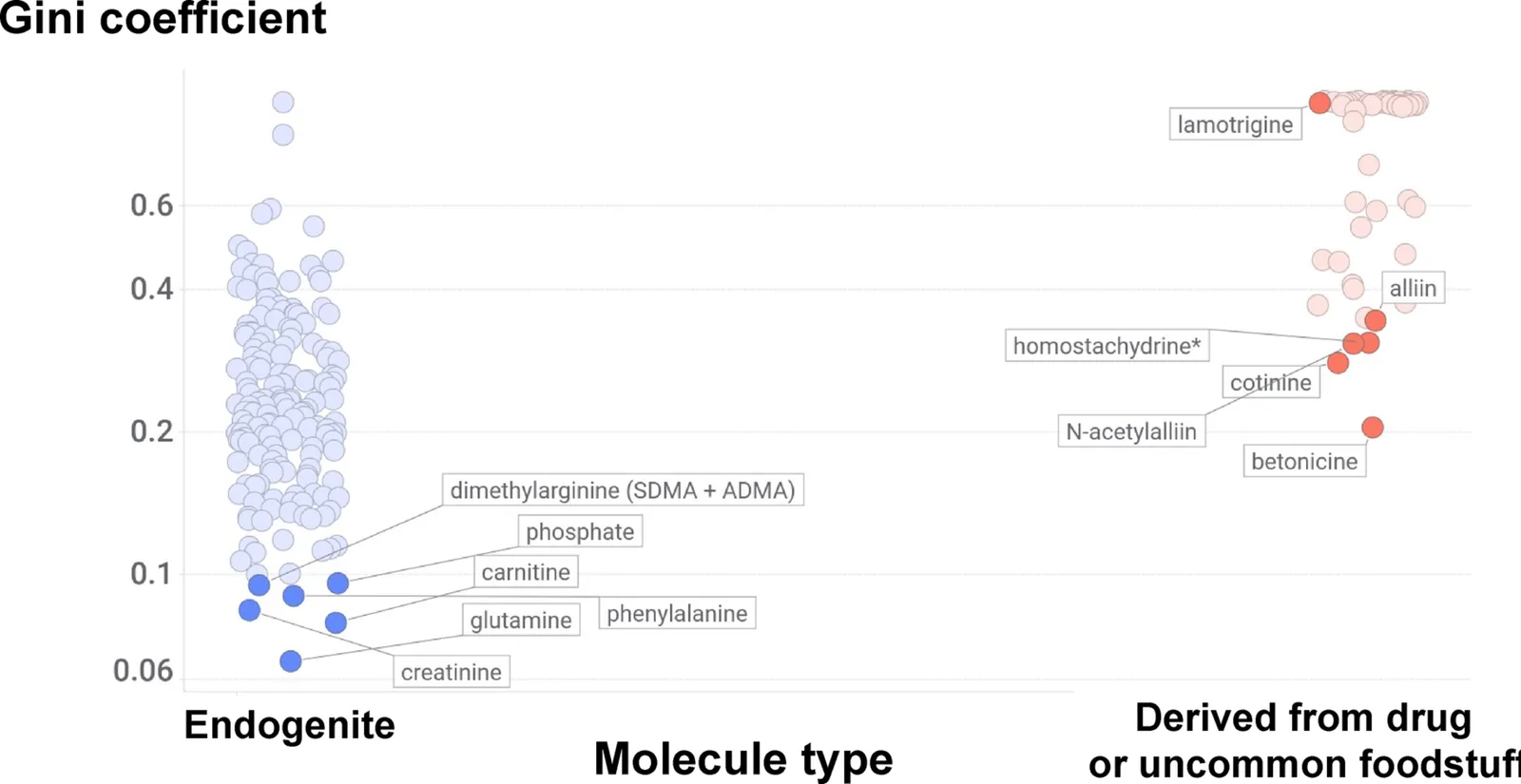
Variation of the Gini coefficient in the ES+ plasma metabolomic dataset Metabolomics Workbench ST002089 from the study of Gillenwater and colleagues (Gillenwater et al., 2021)

It is also of interest to determine which metabolites have unusually low Gini coefficients in the Metabolomics Workbench ST002089 dataset, and the lowest five in each class are labelled in Fig. 3. We can suggest several conclusions from this analysis.

1. The sharp difference in Gini coefficient between the two classes provides a strong hint as to whether a metabolite is or is not a major and regulated player in the endogenous metabolism of the organism. This said, we recognise that, as mentioned above, for reasons explained within the framework of metabolic control analysis (Cornish-Bowden, 1999; Fell, 1992; Heinrich & Schuster, 1996), metabolite concentrations (though not fluxes) can be highly sensitive to changes in the activity of individual enzymes (Kell & Oliver, 2016; Raamsdonk et al., 2001).
2. Glutamine is the endogenous metabolite (endogenite), with the lowest Gini coefficient, with a value (of 0.066, 95% CI 0.063–0.068) that (despite a population of 1125) is in fact considerably lower than any observed in our transcriptomics analyses (O’Hagan et al., 2018; Wright Muelas et al., 2019). This is reasonable as it is a major hub of nitrogen metabolism and virtually by definition, given its Gini coefficient, must be highly regulated. It might be seen as a suitable molecule for normalisation of other metabolite concentrations if there is doubt about variations in the mass or volume of samples in a particular study. The metabolites with the next lowest Gini coefficients were carnitine (0.079, 95% CI 0.075–0.083) and creatinine (0.084, 95% CI 0.080–0.088), while one of many pharmaceuticals (lamotrigine) had a Gini coefficient of 0.99 (95% CI 0.983–0.994). The tight 95% confidence intervals are not surprising in the light of the number of individuals studied.
3. ‘Foodomics’ has been used to identify biomarkers for particular foodstuffs (e.g. (Laghi et al., 2014; Muguruma et al., 2022; Trimigno et al., 2018). Four of the five ‘exogenous’ metabolites are easily identified as being derived from particular foodstuffs (see e.g. (Beckmann et al., 2020; Brennan, 2026)) that may be assumed to be widely consumed in this cohort: alliin and N-acetylalliin from garlic, and homostachydrine and betonicine (C_7_H_13_NO_3_, 4-hydroxystachydrine) from citrus fruits. Cotinine is the major metabolite of nicotine and as a population exhibiting COPD (and as recorded in the original dataset) the vast majority of this cohort were smokers. Consequently, all of this makes sense.

We next assessed a dataset involving ergothioneine and dementia (class AD), also including healthy young (HY) and healthy elderly (HE) individuals (Teruya et al., 2021). Table 2 summarises those data, with the Gini coefficient in the whole sample having a value of 0.325.

**Table 2:** Gini coefficients for ergothioneine in a study (Teruya et al., 2021) of individuals considered either healthy or to have dementia

Group	n	Median (raw abundance)	Gini
All	24	3.00×10^8^	0.325
AD	8	1.629×10^8^	0.209
Healthy Elderly	8	3.938×10^8^	0.280
Healthy Young	8	3.677×10^8^	0.157

**Fig. 4:**
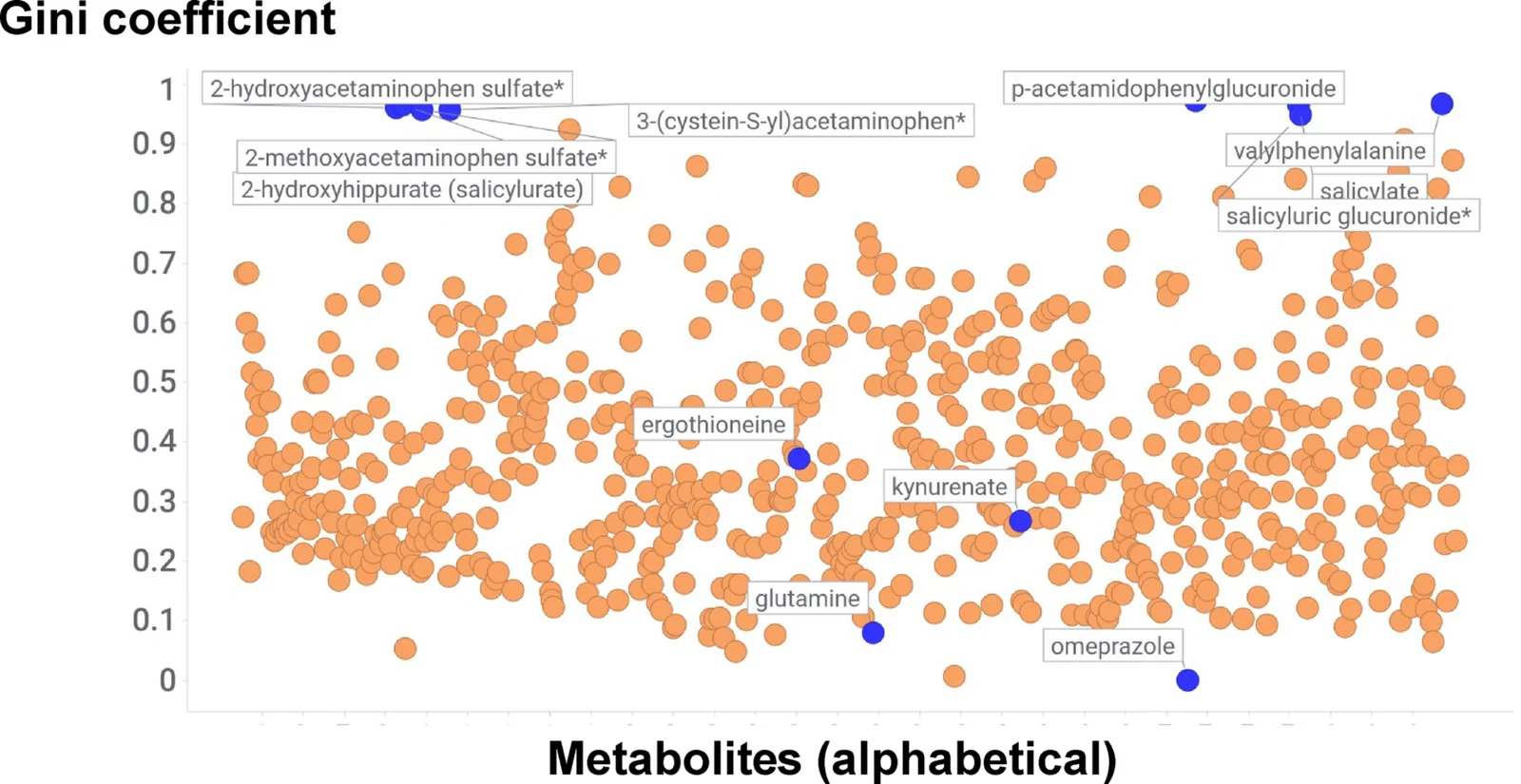
Gini coefficients of 681 metabolites from the dataset at metabolomics workbench ST000975 (Weiner et al., 2012)

Another large metabolomic dataset (Metabolomics Workbench ST000975) consisting of 340 individuals and 681 metabolites present in serum comes from a study (Weiner et al., 2012) of individuals infected (or not) with *Mycobacterium tuberculosis*. Figure 4 shows the Gini coefficients as calculated by us therefrom. Clearly the values closest to 1 are metabolites of the exogenous compounds acetaminophen (paracetamol) and aspirin, plus valylphenylalanine (whose origin is unclear). The median Gini coefficient is 0.358. Ergothioneine (see later) is also labelled and has a Gini coefficient of 0.373. Kynurenic acid, another interesting nutraceutical (Alves et al., 2024) with a mainly endogenous origin, is also labelled (its Gini coefficient is 0.269). The very lowest Gini coefficients seem to be artefacts of the analysis as in the case of the drug molecule labelled ‘omeprazole’ essentially the same value appears in each run. Glutamine again has a very low Gini coefficient (0.08).

**Fig. 5:**
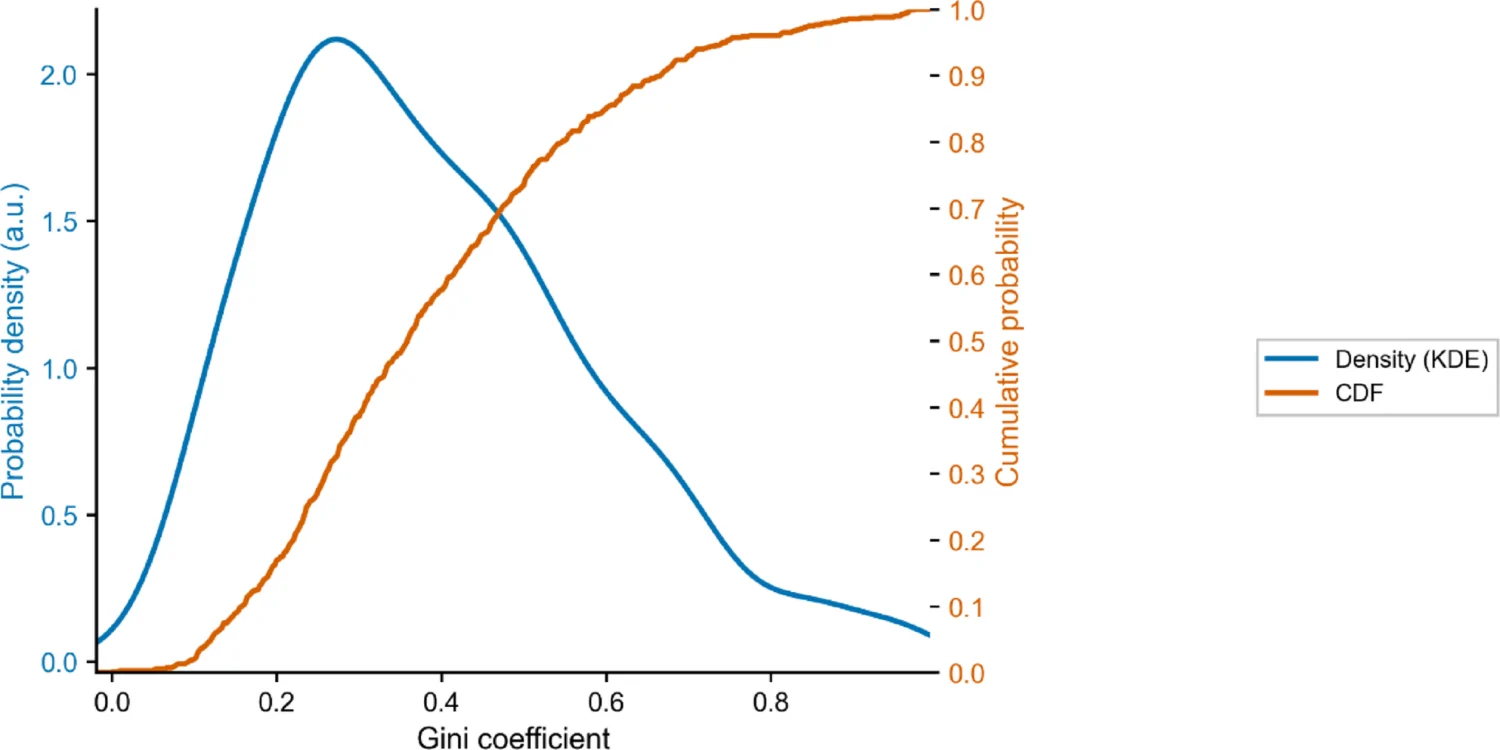
Smoothed probability and cumulative distribution functions for the Gini coefficient data calculated from the ST000975 data displayed in Fig. 4

For convenience, Fig. 5 shows the smoothed probability and cumulative distribution functions for those Gini coefficient data. The curves indicate a wide range of values, implying contributions from both endogenous and exogenous molecules.

**Fig. 6:**
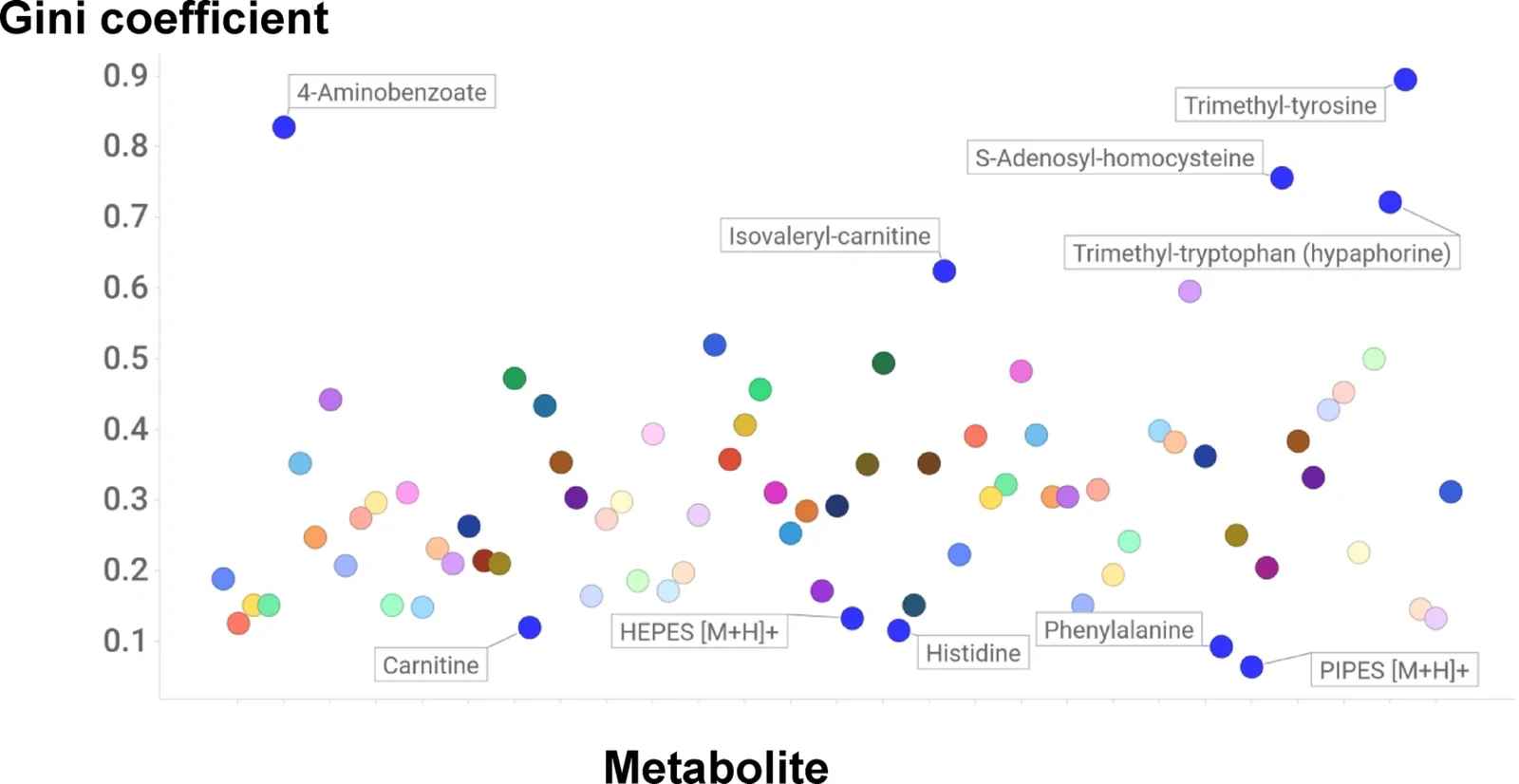
Variation in Gini coefficient for 126 metabolites in a dataset of (Chaleckis et al., 2016)

Chaleckis et al. (2016) studied the variation in some 126 metabolites in plasma (80 in ESI+ mode) between 15 young (29 ± 4 y of age) and 15 elderly (81 ± 7 y of age) individuals. The median plasma ergothioneine levels of the two age cohorts were not significantly different, though the Gini coefficient for ergothioneine in the whole dataset was 0.457. The Gini coefficients for the whole ESI+ dataset (originating from MetaboLights https://www.ebi.ac.uk/metabolights/editor/MTBLS266/files) is given in Supplementary Table 2, while Fig. 6 displays the distribution of ESI+ Gini coefficients, with the top five and bottom five labelled. The median value of the Gini coefficient is 0.297. In this case, apart from the PIPES and HEPES buffers (that are in one sense uninteresting but do provide a useful sanity check), histidine and phenylalanine are lowest, again consistent with the view that organisms strongly regulate their amino acid metabolism.

Table 3 summarises other datasets in which the authors have reported actual concentrations of ergothioneine, Most cluster around median values of ~ 1 µM (229 ng/mL), though there are a couple of cases in which the median is some threefold higher. We recognise that cross-study differences may reflect matrix effects, platform effects, calibration differences, population structure and preprocessing, but our main point here is that the values of the Gini coefficient for ergothioneine are in fact broadly similar.

**Table 3:** Summaries of some studies in which the concentration of ergothioneine and its variation between individuals was measured. Some give concentrations in micromolar, others in ng/mL. For ease, 100 ng/mL ergothioneine ~ 0.436 µM and 1 µM ~ 229.3 ng/mL

Sample	Concentrations	Comments	Reference
Plasma	1.282 µM average in baseline	PK study. Whole blood nearly 10x more.	(Cheah et al., 2017)
Plasma	Median 1.14 µM (0.92–1.45 IQR)	Non-pre-eclamptics. Gini coefficient for all individuals including pre-eclamptics 0.195	(Kenny et al., 2023)
Plasma	Average 3.0–3.3 µM in older and younger individuals	NMR study. More than 10x more in RBCs.	(Nagana Gowda et al., 2025)
Serum	Median 1.01 (IQR 0.78–1.33 µM)	Healthy older individuals (some loss of concentration with age)	(Sotgia et al., 2014)
Serum	Median 0.74 µM (0.39–1.37 IQR)	Gender differences. Gini coefficient 0.376	(Suzuki et al., 2025)
Plasma	Mean 107.4 ± 20.5 ng/ml (0.468 ± 0.089 µM)	Small but very quantitative mass spectrometry study	(Wang et al., 2013)
Plasma	Median and IQR for All 0.762 µM (0.49–1.36)	No Cognitive Impairment I.20 µM (IQR 0.77–1.83) CIND 0.80 µM (IQR 0.53–1.48) Dementia 0.59 µM (IQR 0.40–1.04) Gini : all 0.40, NCI 0.37, CIND 0.39, dementia 0.39.	(Wu et al. 2021a, 2021b)
Plasma	Median baseline 1.15 µM	Increased ~ 3- and ~ 6-fold for 10 mg, and ~ 6- and ~ 16-fold for 25 mg, at weeks 4 and 16, respectively	(Zajac et al., 2025)
Plasma	842.2 ng/mL (3.67 µM) (IQR 657.8–1052.5 ng/mL)	Dementia study in China	(Zhao et al., 2025)

**Fig. 7:**
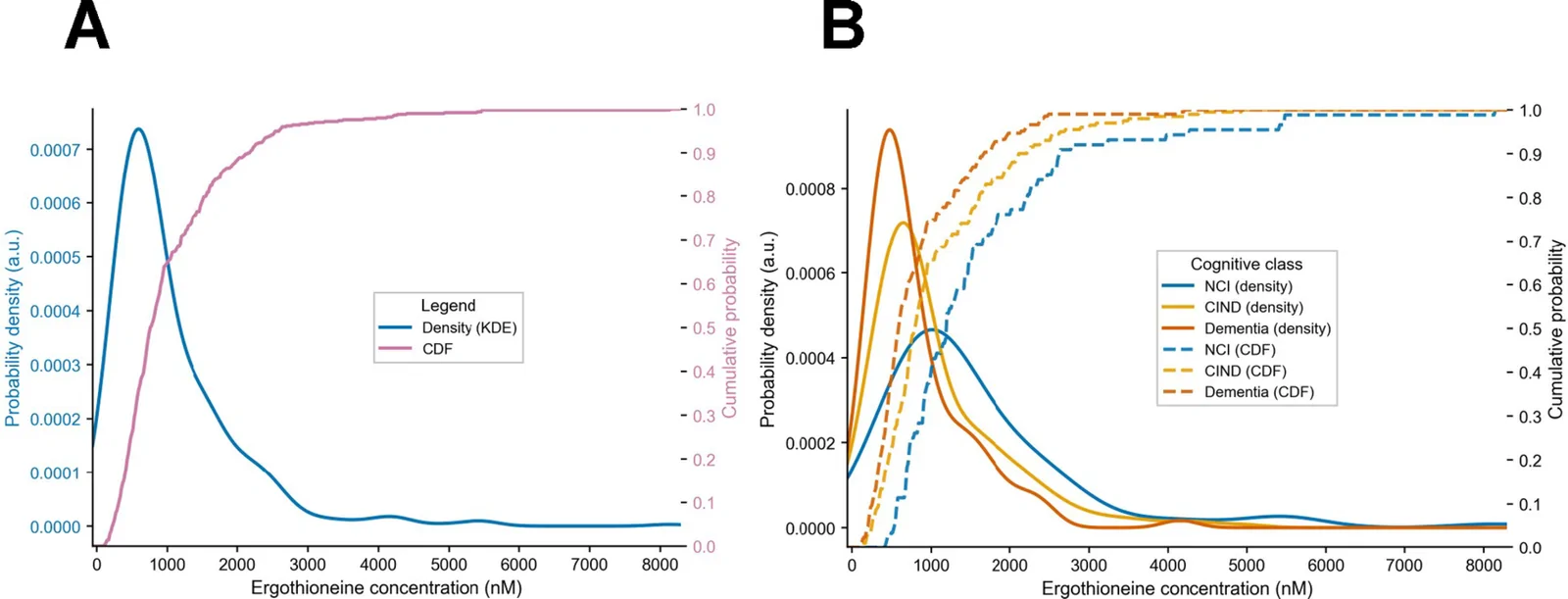
Smoothed probability density and cumulative probability distributions of ergothioneine concentrations in the data of (Wu et al. 2021a, 2021b) for **A** all individuals, **B** the subclasses of NCI, CIND and Dementia defined therein

Wu and colleagues (2021a, 2021b) studied plasma ergothioneine levels in 496 individuals who had either no cognitive impairment (NCI, *n* = 88), cognitive impairment but no dementia (CIND, *n* = 201) and individuals with dementia (*n* = 207). The median value for the NCI individuals was 1.2 µM, almost identical to that of the non-pre-eclamptics in our pregnancy study (Kenny et al., 2023), while those for the other classes were significantly lower (Table 1). The Gini coefficients for ergothioneine (as in (Weiner et al., 2012)) were in the range 0.37–0.4, again consistent with an exogenous origin. The probability density and cumulative distributions for these data (Wu et al. 2021a, 2021b) are shown in Fig. 7, Lower ergothioneine levels are clearly associated with an increased likelihood of cognitive impairment and dementia, consistent with other studies (Cheah et al., 2016; González-Domínguez et al., 2021; Kondoh et al., 2022; Meng et al., 2025; Oka et al., 2024; Teruya et al., 2021; Yau et al., 2024), with these facts and the significant Gini coefficient also indicating the scope for exogenous addition. Were ergothioneine concentrations normally distributed (they are not (Kenny et al., 2023) a Gini coefficient of 0.35–0.4 would equate to a coefficient of variation of ca. 0.8–1 (Roberts et al., 2026).

**Fig. 8:**
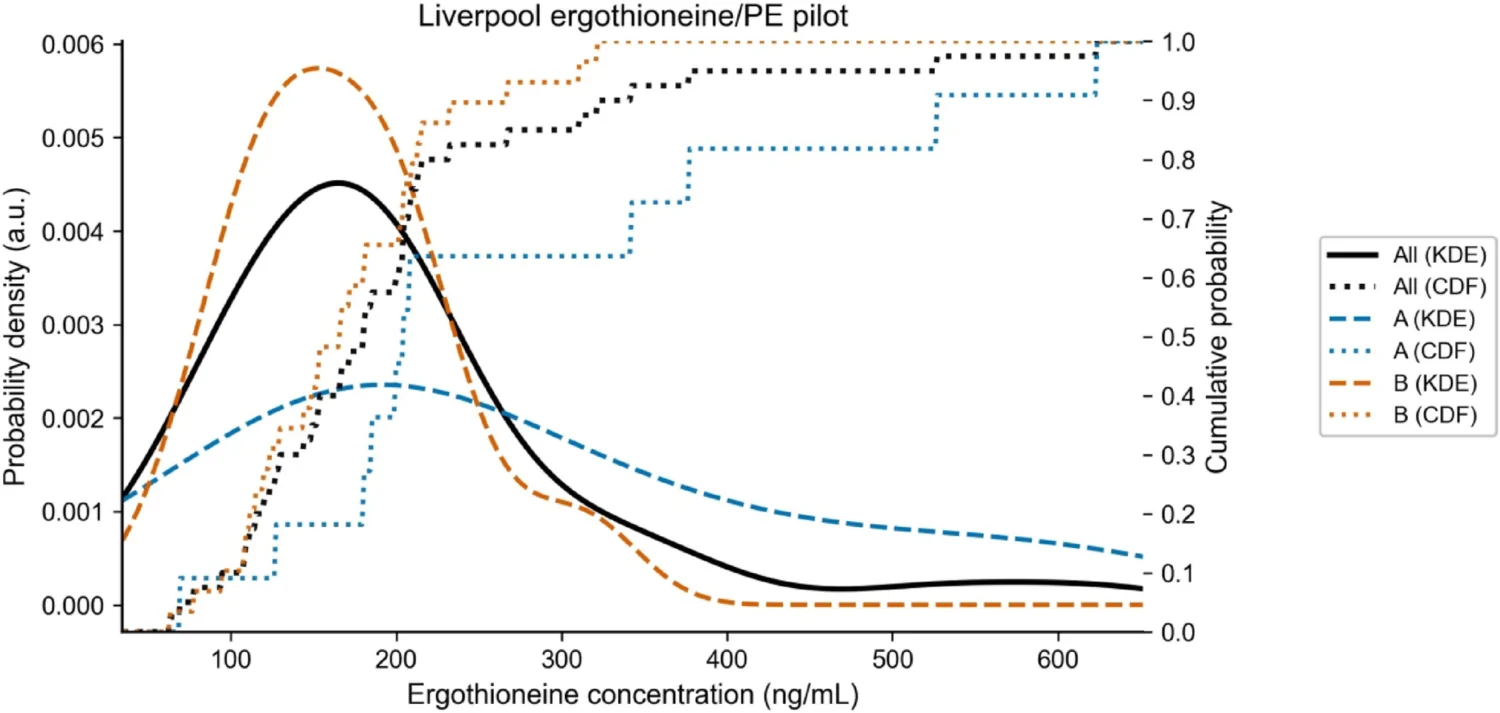
Probability density and cumulative distribution functions for plasma ergothioneine data in a pilot study in Liverpool (Table 4). In the legend, A represent those who developed pre-eclampsia (whether pre-term or term) which B represents those who did not

**Fig. 9:**
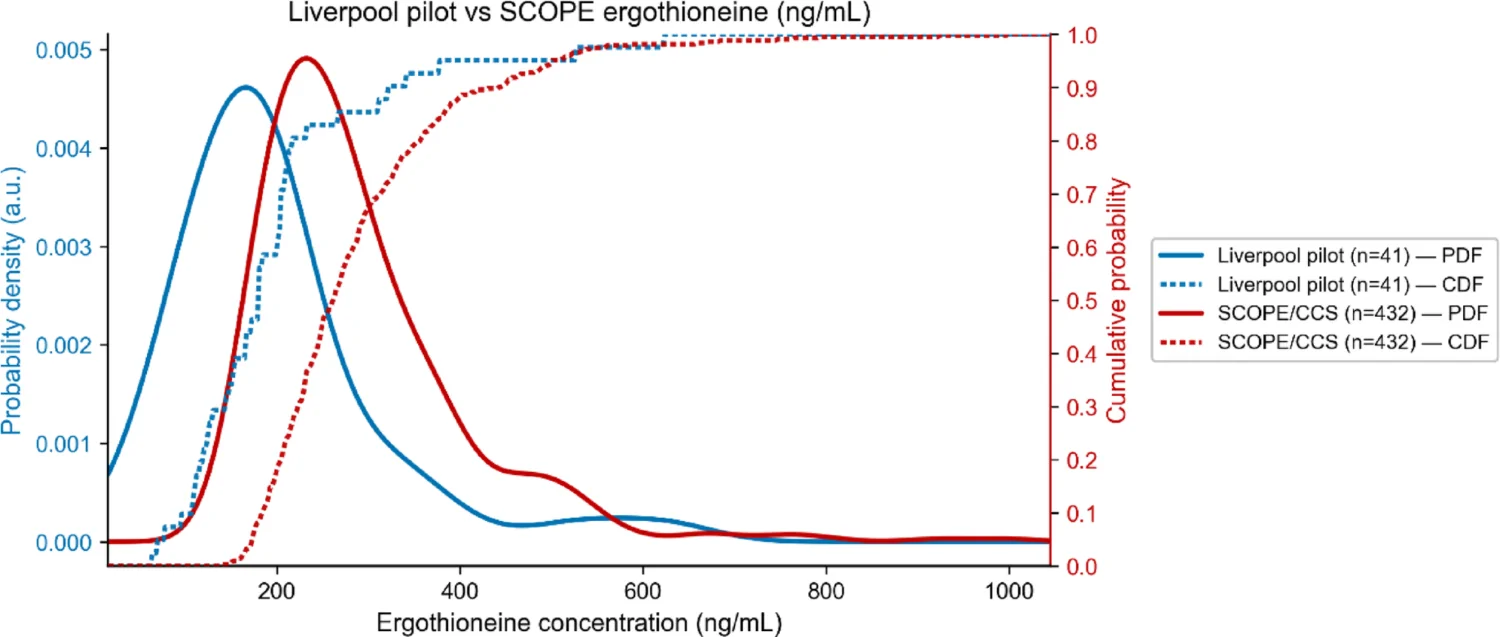
Probability density and cumulative distribution functions for plasma ergothioneine data in 40 participants in a pilot study in Liverpool (Table 4, blue) as compared with those from an earlier study of 432 individuals in the SCOPE study (Kenny et al., 2023)

In our earlier study of ergothioneine and pre-eclampsia using samples from the SCOPE study, where those with high ergothioneine levels were found very much less likely to develop pre-eclampsia, we found a median for all samples of ca. 1.14 µM (0.92–1.45 IQR) equivalent to 261 ng/mL (IQR 211–332 ng/mL). We recently performed a small pilot study in Liverpool, with what initially seemed to be surprising results in that those developing (pre-term or term) pre-eclampsia tended to have slightly higher levels of ergothioneine than those who did not. The deviations for low and high concentration calibration solutions analysed in duplicate at the start and end of the analytical run were; 0.1 µmol.L^− 1^ was reported as 0.12 µmol.L^− 1^ and 5.0 µmol.L^− 1^ was reported as 4.85 µmol.L^− 1^). The calibration approach applied did not use internal standards or a standard reference material and so defines the distribution of concentrations but does not define the highest level of accuracy available. Table 4 and Fig. 8 show the relevant data, while Fig. 9 compares the Liverpool pilot data with those of all participants in the previous study. The metadata are given in Supplementary Table 3.

**Table 4:** Distributions of plasma ergothioneine concentrations in a pilot study of pre-eclampsia in Liverpool

Group	*n*	Median (µM)	Median (ng/mL)	Gini coefficient (95% CI)
Pre-eclampsia	11	0.889	203.9	0.316 (0.18–0.38)
No pre-eclampsia	29	0.722	165.5	0.206 (0.15–0.25)
All	40	0.784	179.9	0.275 (0.19–0.33)

Statistically, this was a somewhat underpowered study in that one class contained only 11 individuals and the other 29, making bootstrapping somewhat hazardous. The Mann-Whitney U value was 0.056, impying that the median values of the two groups (pre-eclamptic and controls) were not significantly different. Two very striking observations do emerge, however. First, the median level of ergothioneine in this study (180 ng/mL) is far below that (261 ng/mL) in the previous SCOPE study (180 ng/mL actually represents just the 9th percentile for the 432 participants in the earlier study (Kenny et al., 2023), as seen most obviously in Fig. 9. Secondly the Gini coefficient for the participants in the Liverpool study is greater than that for the earlier study, but still well below the values typical for ergothioneine in other studies. This could be taken to mean that even if absolute values of ergothioneine concentrations were not available the Gini coefficient, combined with other sociological and demographic data, might give a useful indication of the potential benefits of supplementation.

### Plasma metabolites derived from the gut microbiome

It is an interesting question as to which plasma metabolites derived from the gut microbiome count as ‘exogenous’, though clearly unusual metabolites of foodstuffs would be prime candidates, and it is of interest to assess the Gini coefficients of those that might be solely microbiome-derived. We sought to focus on mass spectrometry-based studies (Dekkers et al., 2022; Jariyasopit & Khoomrung, 2023; Liao et al., 2021). Thus, Dekkers and colleagues (Dekkers et al., 2022) argued that the gut microbiota might explain up to 46% of the variance of individual plasma metabolites. However, the raw data for calculating the metabolite Gini coefficients are not online for any of these three papers. Gini-based ranking might usefully be combined with microbiome-metabolome multi-omics (Mengucci et al., 2022) in future work.

### Gini coefficients of the urinary metabolome

Although our main focus was on plasma/serum, we analysed three datasets from Metabolomics Workbench containing data on the urinary metabolome (Table 5). One hypothesis could be that the Gini coefficient of the urinary metabolome might on average be greater than those of plasma and serum. However, that was not obviously the case (compare Table 5 with earlier plasma/serum data).

**Table 5:** Median Gini coefficients of three datasets obtained from metabolomics workbench

Metabolomics workbench ID	Median Gini coefficient	Comments	Reference
ST001928	0.421	LC-MS. 61 samples, 305 metabolites	(Frankenfeld et al., 2022)
ST001385	0.297	LC-MS. 346 samples, 299 metabolites	Not seemingly published
ST000381	0.381	GC-TOF-MS. 92 samples, 200 metabolites	(Kind et al., 2016)

## Discussion and conclusions

This paper had two main aims. The first was to test the idea that the Gini coefficient might be useful for discriminating metabolites that are tightly regulated (normally endogenous) versus those that are not (potentially having an exogenous origin), and apart from essential amino acids this was clearly the case. We do recognise, as pointed out by a referee, that a Gini coefficient might be raised as a result of analytical errors, missing values not taken into account, and various other metabolic features such as variable pharmacokinetics, as such might be seen as a hypothesis-generating descriptor. This said, a low Gini coefficient cannot be seen as greatly influenced in these ways, and we consider that the Gini coefficient provides a convenient metric for summarising the variation within metabolomics datasets, and we recommend it accordingly. Although this is somewhat arbitrary, we can suggest that metabolites with Gini coefficients below around 0.25 are subject to significant homeostasis or low variation in exogenous supply, while those with a Gini coefficient above around 0.75 are likely to have an exogenous origin and/or be largely unregulated. Molecules in an intermediate range are likely to be exogenous but somewhat regulated, and this was true for most vitamins and the nutraceuticals ergothioneine and kynurenic acid. Overall, we suggest that the Gini coefficient provides a convenient means of describing the extent to which a metabolite varies its concentration over a metabolomic dataset.

Foodomics and nutritional metabolomics studies have shown that NMR- and MS-based profiles can identify biomarkers of food intake in biofluids; such biomarkers may be unevenly distributed across cohorts because they depend on exposure, timing, absorption, microbiome metabolism and clearance. The Lorenz/Gini coefficient may therefore provide a useful complementary descriptor of whether a metabolite behaves more like a tightly regulated endogenous compound or a variable marker of dietary or other exogenous exposure.

A second aim was to study the variation in ergothioneine concentrations in various populations, including in a pilot study of our own on pre-eclampsia in Liverpool. Here, in contrast to an earlier study (Kenny et al., 2023), we were surprised to find no apparent benefits of ergothioneine, until we recognised the very low levels of ergothioneine in this population as compared with those of the earlier study. The value of the Gini coefficient was also rather lower than in almost all other studies of ergothioneine distributions in populations (Table 3), indicating that even if absolute concentrations have not been measured a low Gini coefficient might raise suspicions of a low range and hence potentially inadequate values. The median value of ergothioneine here (179 ng/mL) was equivalent to that of just the 9th percentile in the previous study (Fig. 9, and see (Kenny et al., 2023; Roberts et al., 2026). This could be taken to reflect the substantial health inequalities in Liverpool and elsewhere in the UK (e.g. (Noonan et al., 2016), not least as summarised in mortality statistics (de Haro Moro et al., 2025; Kontopantelis et al., 2018; Taylor-Robinson & Esan, 2025; Walsh et al., 2026; Ward et al., 2025), and if anything makes the case for ergothioneine supplementation in areas such as Liverpool even stronger.

## Supplementary Information

Below is the link to the electronic supplementary material.

Supplementary Material 1

Supplementary Material 2

Supplementary Material 3

## Data Availability

All data supporting the findings of this study are available within the paper and its Supplementary Information.
