# Supplementary material for: The Gini coefficient as a surrogate for the regulability or homeostasis of metabolite concentrations: focus on ergothioneine
Source: Metabolomics. 2026 Sep 16;22(5):151. doi: 10.1007/s11306-026-02534-1 (PMC13582372; doi:10.1007/s11306-026-02534-1)
Supplement: Supplementary file 1 [file 11306_2026_2534_MOESM3_ESM.docx]

| **Participant number** | **Age** | **BMI** | **Ethnicity** | **Gravida** | **Parity** | **Gestational age (in weeks + days) at recruitment** | **Pre-eclampsia (Y/N)** | **Gestational age (weeks+days) at diagnosis of pre-eclampsia** |
| --- | --- | --- | --- | --- | --- | --- | --- | --- |
| BIOPE001 | 28 | 32 | White British | 1 | 0 | 29+1 | Y | 28+1 |
| BIOPE002 | 31 | 40 | Mixed | 1 | 0 | 33+5 | Y | 32+3 |
| BIOPE003 | 36 | 24 | White British | 1 | 0 | 30+2 | Y | 30+1 |
| BIOPE004 | 33 | 36 | White British | 1 | 0 | 34+6 | Y | 29+4 |
| BIOPE005 | 36 | 21 | Black African | 1 | 0 | 36+1 | Y | 36+0 |
| BIOPE006 | 24 | 28 | White British | 1 | 0 | 35+5 | Y | 35+3 |
| BIOPE007 | 35 | 34 | White Irish | 1 | 0 | 38+2 | Y | 36+6 |
| BIOPE008 | 30 | 26 | White British | 1 | 0 | 38+5 | Y | 38+2 |
| BIOPE009 | 32 | 30 | Black African | 2 | 0 | 22+2 | Y | 21+3 |
| BIOPE010 | 35 | 35 | White British | 1 | 0 | 37+5 | Y | 37+4 |
| BIOPE011 | 23 | 20 | White British | 4 | 1 | 28+5 | N |  |
| BIOPE012 | 32 | 22 | White British | 6 | 2 | 35+1 | N |  |
| BIOPE013 | 25 | 25 | White British | 6 | 4 | 35+3 | N |  |
| BIOPE014 | 24 | 32 | White British | 4 | 1 | 28+6 | N |  |
| BIOPE015 | 22 | 26 | Asian | 3 | 2 | 35+1 | N |  |
| BIOPE016 | 36 | 27 | White British | 5 | 3 | 29+1 | N |  |
| BIOPE017 | 28 | 27 | Mixed | 2 | 1 | 36+1 | N |  |
| BIOPE018 | 35 | 29 | Other | 3 | 1 | 30+1 | N |  |
| BIOPE019 | 20 | 34 | Asian-Pakistani | 3 | 1 | 37+0 | N |  |
| BIOPE020 | 34 | 28 | NA | 3 | 1 | 37+2 | N |  |
| BIOPE021 | 35 | 24 | White British | 2 | 1 | 38+5 | N |  |
| BIOPE022 | 24 | 30 | White British | 2 | 1 | 37+5 | N |  |
| BIOPE023 | 27 | 27 | Asian-Bangladeshi | 2 | 1 | 36+3 | N |  |
| BIOPE024 | 33 | 32 | White British | 2 | 1 | 23+1 | N |  |
| BIOPE025 | 36 | 18 | Asian-Pakistani | 2 | 1 | 33+4 | N |  |
| BIOPE026 | 28 | 25 | White British | 3 | 1 | 38+4 | N |  |
| BIOPE027 | 35 | 21 | White British | 2 | 1 | 35+0 | N |  |
| BIOPE028 | 33 | 24 | White British | 3 | 1 | 36+0 | N |  |
| BIOPE029 | 25 | 20 | White British | 2 | 1 | 35+2 | N |  |
| BIOPE030 | 29 | 26 | White British | 3 | 2 | 36+3 | N |  |
| BIOPE031 | 31 | 26 | White-other | 2 | 1 | 33+3 | N |  |
| BIOPE032 | 34 | 20 | White British | 2 | 1 | 32+6 | N |  |
| BIOPE033 | 23 | 28 | White-other | 2 | 1 | 22+6 | N |  |
| BIOPE034 | 39 | 20 | White British | 5 | 4 | 23+0 | N |  |
| BIOPE035 | 31 | 33 | White British | 2 | 1 | 39+1 | N |  |
| BIOPE036 | 25 | 23 | White British | 2 | 1 | 38+0 | N |  |
| BIOPE037 | 40 | 27 | White British | 3 | 2 | 38+6 | N |  |
| BIOPE038 | 34 | 21 | White British | 2 | 1 | 38+0 | N |  |
| BIOPE039 | 25 | 26 | White British | 3 | 1 | 30+4 | N |  |
| BIOPE040 | 21 | 33 | White British | 3 | 2 | 30+3 | Y | 36+5 |
